# ASO Author Reflections: A Systematic Review of Factors Affecting Quality of Life After Cytoreductive Surgery with Hyperthermic Intraperitoneal Chemotherapy

**DOI:** 10.1245/s10434-020-08435-4

**Published:** 2020-04-07

**Authors:** B. L. Van Leeuwen, S. Kruijff

**Affiliations:** Department of Surgery, University of Groningen, University Medical Center Groningen, Groningen, The Netherlands

## Past

Only 3 decades ago, patients with peritoneal metastasis, either metachronous or synchronous, were considered incurable and suitable only for palliative treatment. Back then, if left untreated, peritoneal metastasized patients had poor prognosis, high morbidity, and reduced quality of life (QoL).^1^ Fortunately, a curative-intent treatment option arose: cytoreductive surgery (CRS) combined with hyperthermic intraperitoneal chemotherapy (HIPEC). This suddenly gave patients with resectable peritoneal metastasis (PM) the option of undergoing a potentially curative treatment. CRS/HIPEC combines surgical removal of all macroscopically visible disease with perfusion of the abdominal cavity with heated chemotherapy to eradicate residual microscopic disease. After its introduction, cumulative scientific evidence seemed to illustrate improved survival outcomes when compared with systemic chemotherapy alone.^2^ If selected carefully, e.g., without distant metastases, aggressive CRS/HIPEC seemed to be a potentially curative treatment for 30–40% of patients. Unfortunately, today, this invasive procedure is still accompanied by a high treatment-related mortality of 0–8%, a grade 3–4 morbidity of 18–52%, and a negative impact on QoL of patients up to 1 year after.^3^ Since the majority of patients undergoing CRS/HIPEC will not be cured by this procedure, the high morbidity rates are an ongoing concern.

## Present

In literature, published systematic reviews regarding QoL after CRS/HIPEC concluded that patients, after experiencing a significant decrease in QoL, usually return to baseline QoL levels within 12 months after surgery.^3^,^4^ However, a high proportion of patients lost to follow-up in these studies probably led to underrepresentation of the most frail patients in these cohorts. Also, most of these reviews rely on limited literature searches, seldomly reporting a wide range of QoL domains.^3^,^4^ Often little consideration was given to the specific determinants of QoL after CRS/HIPEC such as stoma placement, disease recurrence, and drop-out rates. Therefore, this systematic review analyzed the primary outcomes reflecting the short-term (< 6 months) and medium-term (6–12 months) determinants of QoL after CRS/HIPEC. Secondary outcomes were QoL and reported symptoms over time.^5^ We included 14 studies that used 12 different questionnaires, and reported data were collected for 1556 patients (dropout < 50% in 4 studies). Overall, collected data showed indeed a diminished QoL within 3 months after surgery but with a recovery to baseline by 12 months. For the specific determinants, we noted that QoL was negatively influenced by higher age, female sex, prolonged operation time, extensive disease (high PCI), residual disease, adjuvant chemotherapy, postoperative complications, stoma placement, and recurrent disease.^5^

## Future

Although this review provides a structured literature oversight reflecting the QoL of patients after having undergone CRS/HIPEC in the first postoperative year, results should be interpreted with caution. Data from the studies collected only applied to patients who were fit enough to remain in the QoL analysis. In fact, only 4 of the 14 studies based their data on more than 50% of their primary enrolled population. Only a small fraction of the missing patients could be explained by deaths not related to cancer, but usually more often due to weakness, disease recurrence, and significant symptomatology.^6^ This makes it reasonable to conclude that QoL may be overestimated in these analyses. Therefore, in the future, when assessing QoL after CRS/HIPEC, we will have to focus more on study designs describing the profound experiences of CRS/HIPEC patients. If we truly want to learn more about the lives of our patients after having undergone CRS/HIPEC, it is essential that patients receive detailed and honest counseling at different time points during their care trajectory. Also, with this in the back of our minds, we should select increasingly on the potential risk of treatment-related diminished QoL rather than solely on the potential survival benefit. Therefore, in the future, high-quality QoL research should be of high importance for all CRS/HIPEC centers to improve the lives of their future peritoneal metastasized patients.

After all, for most patients, the HIPEC procedure is part of the final stages of their life. A period where quality of life is of great importance.
